# Incomplete removal of basal cell carcinoma: what is the value of further surgery?

**DOI:** 10.1007/s10006-012-0348-3

**Published:** 2012-08-07

**Authors:** Shiralee S. Patel, Sandeep H. Cliff, Peter Ward Booth

**Affiliations:** Oral and Facial Department, East Surrey Hospital, Canada Avenue, Redhill, Surrey RH1 5RH UK

**Keywords:** Basal cell, Positive margin, Residual tumour

## Abstract

**Introduction:**

Surgical management of skin cancer is an important part of modern maxillofacial surgery. The common tumours treated are squamous cell carcinoma, malignant melanoma and some benign lesions, but the largest group of tumours are the basal cell carcinomas. Although only locally aggressive, if they are not completely removed, recurrence may occur and be troublesome, especially in the head and neck. Even in this region, incomplete excision is uncommon, less than 20 %, but management of positive margins remains controversial. This review evaluates the effectiveness of a further surgical intervention after a positive margin.

**Materials and methods:**

A retrospective audit was undertaken to determine the rate of positive margins within the unit and subsequently the percentage of residual tumour found in any secondary excisions.

**Results:**

The results show that in a sample of 247 patients, 11 % had positive peripheral margins. A second excision only showed that 36 % had any evidence of residual tumour.

**Discussion:**

The study raises the question of the value of further surgery. Finally, the authors suggest a more focused approach to the finding of a positive margin before the patient is offered more treatment.

## Introduction

Basal cell carcinoma (BCC) is a very common skin tumour which is usually slow-growing and benign in nature, with metastasis being exceptionally rare. A variety of treatment methods have been described: surgery, radiotherapy and chemotherapy, used topically or in conjunction with photodynamic therapy, curettage and cryosurgery. The type of treatment should be based on the patient's medical history, type, size and site of the tumour, but may also depend on the experience of the clinician who first assesses the patient.

For those patients who are treated by surgical excision, very high success rates, in terms of elimination of the tumour, should be expected. The British Association of Dermatologists (BAD) estimate that if the tumour were removed with a clinical margin of 3 mm, it would be expected that, microscopically, the tumour would be adequately excised in 85 % of cases [1]. Other histopathological factors may mitigate against successful removal. For example, an aggressive lesion with perineural spread may produce skip lesions outside the excision margins. For certain types of BCC, such as morphoeic, infiltrative or multifocal lesions, the peripheral margins, and therefore positive margins, are more difficult to identify, so the risk of recurrence is more likely. In such difficult cases, Mohs dermographic surgery, an interactive histological checking of the margins, may be required.

## Material and method

### Organisation of the service

The maxillofacial unit is part of the team managing skin cancer. The local team consists of dermatologists, surgeons, pathologists and support nurses. This team integrates into a regional cancer team with other units and additionally includes oncologists and radiotherapists. BCCs would not normally be discussed at the regional team level. Since it has been reported that the diagnostic success of dermatologists is much greater than non-dermatologists, the dermatologist sees all patients first, and the surgical cases are referred on to the surgeons, the majority going to maxillofacial surgery.

### Organisation of the study

In this study, we looked at all cases of skin cancer removed by maxillofacial surgery for the period of January–December 2009 undertaken by one surgeon. During this time, not all the cases were sent to the same histopathology laboratory, and so the study was reduced to include only those sent to the Surrey & Sussex Histopathology Department, and which were histologically proven to be BCCs.

The patients were identified from the database by the surgeon's name and then by the histopathological diagnosis BCC. The pathology reports were then reviewed retrospectively.

Of the 792 patients operated on by the surgeon, only 247 BCCs were studied. These cases were only selected on the basis that the histopathological examination was carried out at the Surrey & Sussex Pathology Department. There was no clinical bias, which would affect the referral to this department. All the surgery was undertaken by one surgeon, regardless of where the histology was finally reviewed.

## Results

The total positive-margin rate for the 792 patients was 10 %, but of the 247 patients in the study, it was 11 %. Table 1 summarises the results. Of the 247 cases which were identified for inclusion in the audit, 232 excisions were from the head and neck region, 11 from the limbs and 4 from the trunk. In total, 29 (11.7 %) had positive margins, and 96 % of these were found on the head and neck (11 % of all specimens).

**Table 1:** Summary of results

	No. of total sample	% of total sample	No. of positive margins	% of total sample	% of positive margins
Head and neck	232	94	28	11	96
Trunk	4	2	1	0.7	4
Limbs	11	4	0	–	–
Total	247	100	29	11.7	100

**Fig. 1:**
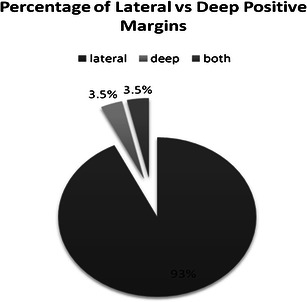
Percentage of lateral vs. deep margins

Figure 1 shows the percentage of lateral margins found to be involved compared to deep margins. In the majority of cases, the lateral margin was involved.

**Fig. 2:**
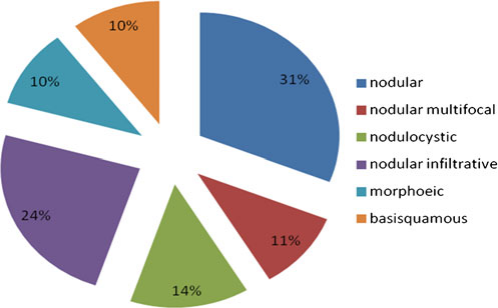
Types of BCC found in primary excisions with histologically positive margins

The main type of BCC (Fig. 2) found in the positive-margin group was nodular, followed by nodular infiltrative; in a small number of cases, morphoeic and basosquamous types were found. As the specimen type was not recorded in all cases, it is difficult to determine whether this is a significant factor or not.

**Fig. 3:**
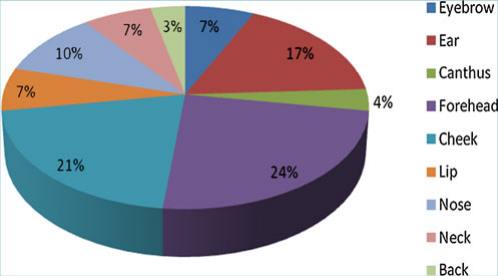
Chart showing sites of origin for positive-margin specimens

Figure 3 shows the location, by percentage, of all specimens with positive histological margins. The forehead and cheek account for almost half of specimens with positive margins.

Of the 29 margin positive excisions, 22 underwent further surgery at a later date and eight (36 %) of these secondary excisions contained residual tumour. Of the seven patients who chose not to undergo further surgery, three have been lost to follow up and four show no evidence of recurrence 18(+) months post primary excision.

## Discussion

These results show that the positive-margin rate is within the expected range of published data. Studies from current literature suggest rates of positive margins ranging from 4 to 16.6 % [2–7]. The BAD recommendation suggests that a 3-mm margin of excision will give an 85 % “clear” margin rate [1]. These figures are above that at 11.7 % positive-margin rate. It is interesting to note the slightly different rates seen in the two samples, the overall cases in 2009 (10 %) and the 247 (11.7 %) cases examined at Surrey & Sussex Pathology Department. This is most likely due to the size of the sample rather than any local variation.

The numbers of cases with positive margins is, as expected, small. It is therefore difficult to draw conclusions as to the cause of the positive margin. In this group, it did not appear to be related to site nor BCC type, but the numbers are too small for any statistical analysis. The literature, however, suggests that head and neck cases have a higher positive-margin rate, as does the canthal area within the head and neck [2, 3, 5]. It is assumed that this is due to the difficulty in removing a relatively benign tumour without wishing to damage important anatomical structures. Similarly, the literature suggests greater difficulty in complete removal of BCCs of certain clinical types, morphoeic, infiltrative and multi-focal [2]. This reflects the difficulty of clinically identifying the margin and the diffuse spreading nature of these lesions. Again, this study showed none of these issues to be important.

In addition, the study confirms the impression that re-operating, even soon after the primary surgery, will produce very few cases where tumour still remains visible, with 64 % being tumour-free. This could be explained by caution on the behalf of the histologists to call a positive margin when in reality it was a close margin. Some have suggested that the surgery stimulates the immune system to eliminate the remaining tumour cells, an event not seen in other tumours, but theoretically possible [8].

With regards to secondary excisions, the BAD guidelines [1] suggest that residual tumour is present in 45–54 % of specimens, whilst other current literature reports a range of 28–54 %. Our figure of 36 % falls within this range [2–7].

Whatever the reason, as surgeons, we need to be able to advise our patients as to the most likely outcome of further surgery. For some patients, who understand the tumour to be benign in its behaviour, justifying the prospect of further surgery is hard as they will be informed that the likely outcome is that there will be no tumour present at the second operation. Other patients, however, will not want the anxiety of possible recurrence.

A more logical approach in view of these findings might be as follows:

- Review the histology, in particular
  - Is the margin “positive” or “close”?
  - Is the deep margin clear?
  - Is it histologically an aggressive lesion?
  - Are the anatomical site, and the positive-margin site, important enough to be considered “high risk”? For example, positive against the medial canthal ligament.

The surgeon could, thus, give the patient more useful information. For example, with a peripheral margin, which is close on the temple with no evidence of perineural spread and the patient understands the situation, it may be worth asking the patient and his family doctor to watch the area. Alternatively, for an aggressive lesion which is large and in the medial canthal area, it would be more appropriate to advise the patient that more treatment is indicated.

Another raft of treatment options, to those patients with positive margins, is of course, also available. For example, radiotherapy may be considered; this is obviously unsatisfactory in some sensitive areas particularly close to the eye, where there is a risk of dry eye or cataract formation, but should be considered, although it does, of course, have the disadvantage of not providing an update of the histology. Other treatments used for primary treatment may also be considered, but like these alternative treatments, since they were not considered to be appropriate initially, these reservations presumably still remain.

It has been suggested that, for those types of BCC which have been shown to be more at risk of recurrence, or indeed a positive margin, it may be appropriate to consider the use of Mohs micrographic surgery in the first instance. The difficulty can sometimes be a case selection for this type of surgery in the primary instance as there was no significant difference found in positive-margin rates between sub-types of BCC in this study. An interesting randomised control trial found that there was no significant difference in recurrence of primary facial basal cell carcinoma between conventional surgical excision and Mohs surgery, but for recurrent facial BCC, treatment via Mohs surgery resulted in significantly fewer recurrences [9]. One other factor to consider when performing Mohs surgery is the time factor—as each case can take 2 h to complete and therefore has a much more limited use.

The previously mentioned limitations of this study lie in the retrospective nature of the audit, and it would be interesting to conduct a more prospective analysis, ideally a randomised controlled trial to gain results which may show more statistical significance.

## Conclusion

This review shows the positive-margin rate to be within the expected range for head and neck BCCs. The study, however, shows a very low presence of tumour in the second resection, questioning the value of further surgery. The authors therefore advocate a more focused approach before offering the patients more surgery. There should be more determination to ensure that it is a positive margin and not a close margin. If the histological features of the lesion are benign and the anatomical site is not a high-risk site, then a wait-and-watch approach may be more justified.
